# 1269. Prospective Evaluation of a Machine Learning Model that Guides Indication for Antibiotics in Lower Respiratory Tract Infections

**DOI:** 10.1093/ofid/ofad500.1109

**Published:** 2023-11-27

**Authors:** Sarah Tang, Maciej Piotr Chlebicki, Shimin Jasmine Chung, De Zhi Chin, Daniel Chang, Andrea Kwa

**Affiliations:** Singapore General Hospital, Singapore, Not Applicable, Singapore; Singapore General Hospital, Singapore, Not Applicable, Singapore; Singapore General Hospital, Singapore, Not Applicable, Singapore; Singapore General Hospital, Singapore, Not Applicable, Singapore; SingHealth, Singapore, Not Applicable, Singapore; Singapore General Hospital, Singapore, Not Applicable, Singapore

## Abstract

**Background:**

Over-prescribing of antibiotics for suspected acute bacterial lower respiratory tract infections (abLRTI) is prevalent. A classification model that can identify instances when antibiotics should be initiated or withheld was previously developed with promising results (80% accuracy). This, however, was based on a retrospective dataset. We conducted a prospective evaluation of this model in the hospital antimicrobial stewardship unit (ASU) to validate its performance and compare its effectiveness against current ASU strategies.

**Methods:**

Inpatients audited by the hospital ASU between 10 January and 23 March 2023 (twice weekly) were enrolled into this study. Relevant patient information (e.g., clinical notes, radiology reports, vital signs) was extracted from our hospital’s electronic data repository for model inference. The presence of abLRTI as determined by the model, the patient’s primary care team and ASU review were compared. Cases that saw differing opinions between any of the three operators were subsequently reviewed by an independent Infectious Diseases physician, who provided the ground truth for that case.

**Results:**

A total of 851 cases were included, of which 235 (27.6%) had true abLRTI. The model was most accurate in determining the presence versus absence of abLRTI (734/851, 86.3%), while ASU review and primary care team were correct in only 698 (82.0%) and 677 (79.6%) cases respectively. Primary care team demonstrated greatest sensitivity in the suspicion for abLRTI (91.9%); unfortunately, it also over-diagnosed abLRTI in 155 (42%) patients. Among these false-positive cases, the model rightly rejected presence of abLRTI in 126 (81.3%) patients whereas only 66 (64.1%) of them were identified by ASU. ASU outperformed the model in having a lower false-negative rate when abLRTI is suspected (17.3% vs. 22.0%).

**Conclusion:**

The model displayed consistent performance in a prospective cohort. Findings support its value in guiding decisions on antibiotic initiation for suspected abLRTI. Deploying this model to augment current ASU capabilities should be strongly considered.

**Disclosures:**

**All Authors**: No reported disclosures

